# Molecular investigation reveals three hemotropic mycoplasmas in cats and three tick species in China

**DOI:** 10.3389/fvets.2025.1522904

**Published:** 2025-01-30

**Authors:** Hongfei Shi, Guoguang Li, Dandan Li, Hongyue Zhai, Shidong Ji, Yun Hu, Long Wang, Lunguang Yao

**Affiliations:** ^1^Henan Provincial Engineering and Technology Center of Animal Disease Diagnosis and Integrated Control, Henan Provincial Engineering Laboratory of Insects Bio-reactor, Nanyang Normal University, Nanyang, China; ^2^Henan Province Engineering Technology Research Center of Animal Disease Control and Prevention, Nanyang Vocational College of Agriculture, Nanyang, China

**Keywords:** cat, *Haemaphysalis longicornis*, *Rhipicephalus microplus*, Candidatus Mycoplasma haemobos, *Rhipicephalus sanguineus sensu lato*

## Abstract

To date, the primary hemoplasmas that infect cats include *Mycoplasma haemofelis*, *‘Candidatus* Mycoplasma haemominutum*’*, and *‘Candidatus* Mycoplasma turicensis*’*. In addition, other hemoplasmas *Mycoplasma* species have also been identified in cats. In central China, no infections or potential vectors with hemotropic mycoplasmas have been recorded in cats. To elucidate the prevalence of hemotropic mycoplasmas in both cats and parasitic ticks, this study investigated the occurrence of hemotropic mycoplasma infections in ticks and cats. A total of 78 blood samples were collected from both anemic and healthy cats, along with 284 ticks from the cats’ body surfaces and 356 ticks found in the surrounding environment. Following the morphological and molecular identification of ticks, all samples were screened for pathogens using PCR detection and sequence analysis. The results indicated the presence of 392 *Haemaphysalis longicornis*, 152 *Rhipicephalus microplus*, and 76 *Rhipicephalus sanguineus sensu lato* in cats and their surrounding environment. Molecular detection revealed the amplification of 156 *‘Ca.* M. haemominutum*’*, 96 *‘Candidatus* Mycoplasma haemobos*’*, 41 *M. haemofelis*, and 64 *Rickettsia felis*-positive amplicons from both cats and ticks. Notably, when comparing the infection rates of *‘Ca.* M. haemobos*’* in the environment group, no significant differences were observed in the infection rates among the three tick species from anemic or healthy cats (*p* > 0.05, *α* = 0.05). Furthermore, sequence analysis of ‘*Ca.* M. haemobos’ indicated two novel sequence types that were most closely related to an isolate from buffalo in China. In conclusion, in this study, in addition to *‘Ca.* M. haemominutum*’* and *M. haemofelis, ‘Ca.* M. haemobos*’* was first detected in cats. ‘*Ca.* M. haemominutum’ appears to be associated with anemic syndrome in cats, while further research is needed to explore the relationship between ‘*Ca.* M. haemobos’ and clinical signs in felines. Additionally, these three hemotropic mycoplasmas were also found in three species of ticks, and transmission experiments are required to investigate the capacity of these ticks to transmit hemoplasmas *Mycoplasma* among animals.

## 1 Introduction

Hemotropic mycoplasmas are small unculturable bacteria that attach to erythrocytes (1). *Mycoplasma haemofelis* (2), *‘Candidatus* Mycoplasma haemominutum*’* (3), and *‘Candidatus* Mycoplasma turicensis*’* (4) are the three main hemoplasmas that infect cats. However, an emerging hemoplasma called *‘Candidatus* Mycoplasma haematoparvum like*’* (5) has been detected in cats in the United States (5), Spain (6), and Japan (7). Intriguingly, in Spain, *Mycoplasma wenyonii* (8), which is primarily associated with cattle infection, was also found in a cat (9). Furthermore, two distinct sequence types of previously undescribed hemotropic mycoplasmas were identified in 15 European wild cats in Bosnia and Herzegovina (10). These findings suggest that cats could be infected by hemoplasmas typically infecting other hosts.

*‘Candidatus* Mycoplasma haemobos*’* (11) is an emerging pathogen that was first detected in cattle (*Bos taurus*) in Japan. Later on, this pathogen was found to infect a diverse range of hosts, including water buffalo (*Bubalus bubalis*) (12), red deer (*Cervus elaphus*) (13), fallow deer (*Dama dama*) (13), roe deer (*Capreolus capreolus*) (13), goats (*Capra aegagrus hircus*) (14, 15), sheep (*Ovis aries*) (14), and dogs (*Canis*) (16). These natural infections are frequently accompanied by anemia (11, 14, 17), transient fever (14), lymphadenopathy (11, 17), anorexia (18), lack of appetite, and decreased milk production (18, 19). To date, natural infections of *‘Ca.* M. haemobos*’* have been reported worldwide, including in Africa (20, 21), Asia (14, 22–25), Europe (13, 26, 27), North America (28), and South America (12, 29). In our previous studies conducted in Henan Province, *‘Ca.* M. haemobos*’* was detected in goats, sheep (14), dogs (16), and cattle (15) on backyard farms. Notably, *Rhipicephalus microplus* and *Haemaphysalis longicornis* ticks infesting the body surface of these animals were also found to carry *‘Ca.* M. haemobos*’* (14–16). Furthermore, *R. microplus* ticks have been implicated as vectors and reservoirs in the transmission of *‘Ca.* M. haemobos*’* (30). On these backyard farms, not only dogs but also cats, which are housed for catching mice and allowed to roam freely, share the same living spaces with infected animals. Given that *R. microplus* and *H. longicornis* can parasitize goats, sheep, dogs, and cats (31–34), it remains uncertain whether cats in this region could become infected with *‘Ca.* M. haemobos*’* through tick infestation.

In China, *Ca.* M. haemominutum was first detected in cats in Guangdong province in 2009 (35), followed by its detection along with *M. haemofelis* and *Ca.* M. turicensis in cats in Shanghai in 2017 (36). To date, no infections with hemotropic mycoplasmas have been recorded in cats in central China. Given this backdrop, this study aimed to focus on the occurrence of *‘Ca.* M. haemobos*’* infections in cats and parasitic ticks in the Henan Province, central China. In addition, due to similar anemia symptoms exhibited in cats, other pathogens such as *Ca.* M. haemominutum, *M. haemofelis*, *Ca.* M. turicensis, *Rickettsia felis* (37), *Anaplasma*, *Hepatozoon*, *Babesia*, and *Theileria* (9) were also included in the investigation.

## 2 Materials and methods

### 2.1 Animals, blood, and tick sample collection

From April 2023 to October 2023, during the peak season for *‘Ca.* M. haemobos*’* infections and tick activity in southern Henan Province (112°38′ ~ 113°24′ E, 33°04′ ~ 33°37′ N), the region is characterized by a diverse topography that includes mountains, hills, and flat or gently rolling plains, situated within the subtropical continental monsoon zone. To address the research question, 19 backyard farms affected by *Ca.* M. haemobos*’* were selected as the study site. Previous research documented infections of *‘Ca.* M. haemobos*’* in goats, sheep, dogs, and cattle on these farms (14–16). Subsequently, investigations were conducted on cats, parasitic ticks, and ticks found in the environment at these locations. In total, 78 EDTA-anticoagulated blood and serum samples were collected from the femoral vein of the cats. Among the sampled animals, some exhibited clinical signs such as pale oral mucous, conjunctival infection, and hematuria. In addition, all ticks found on the skin surface of each cat were collected, resulting in a total of 284 ticks. These ticks were treated individually, following the methodology established in a previous study (14). Furthermore, questing ticks (*n* = 356) in the environment were collected using the drag–flag method (38), using a white cotton flannel cloth (1.2 m × 1 m). The flag was systematically dragged over low vegetation (1 ~ 30 cm in height) near the edges of paths and the borders of dense vegetation.

### 2.2 Blood examination

All blood samples underwent an initial examination as follows: the partial whole blood samples were analyzed using an automated hematology analyzer (DF55Vet, Dymind, China), and the results were compared to established reference ranges (red blood cell (RBC): 6.54–12.20; hematocrit (HCT): 30.30–52.30; hemoglobin (HGB): 9.80–16.20) in accordance with the provided instructions and the reference (39). Subsequently, based on the clinical symptoms documented earlier, the cats involved in this study were categorized into two groups. The remaining blood samples from the cats were preserved at −80°C for future analysis.

### 2.3 Tick morphological identification

All adult tick samples were initially identified using morphological and taxonomic identification methods (40). Hard ticks and soft ticks were differentiated at the family level based on the position of the anal groove. Subsequently, at the genus level, identification was achieved by examining the morphology of the gnathosoma. Within the same genus, ticks exhibit considerable morphological similarity, necessitating the identification of distinguishing features such as the shape, color, and patterns of the scutum; the configuration of the spiracular plates; the presence of stripes on the tarsus; and the characteristics of the pulvilli. Finally, sexual dimorphism was assessed by comparing the size of the scutum between males and females (41).

### 2.4 Primer selection, blood and tick DNA extraction, and pathogen screening and sequencing

The blood samples obtained from cats were subjected to DNA extraction using the EasyPure Blood Genomic DNA Kit (TransGen Biotech, China) according to the manufacturer’s instructions. Each tick sample was homogenized in 1 mL of phosphate-buffered saline, then placed on sterile filter paper to dry, and further put into tubes prefilled with ceramic beads (MagNA Lyser Green Beads, Roche, USA). A volume of 400 μL of PBS was added to each tube, and incubation was carried out for 5 h. After the tick bodies and scuta had softened, the tick tissues were homogenized at 7,000 rpm for 90 s by the MagNA Lyser Instrument (Roche, USA), after which 200 μL of each homogenate underwent DNA extraction utilizing the Universal Genomic DNA Kit (TIANGEN, China). The extracted genomic DNA was eluted in DEPC-treated water and subsequently used as a template in PCR reactions. For molecular identification, the conserved 12S rRNA gene of tick species was selected for amplification using primers T1B and T2A (42). In alignment with previous research (43), a specific pair of primers (H-MYC-F/R) was selected to identify potential hemotropic mycoplasmas. These primers have previously demonstrated efficacy in amplifying the partial 16S rRNA gene of various species, including *M. haemofelis*, *Ca.* M. haemominutum, *M. haemocanis*, *Ca.* M. haematoparvum, *Mycoplasma ovis*, *Candidatus* Mycoplasma haemovis, *M. wenyonii*, and *‘Ca.* M. haemobos*’* (14–16, 43). Furthermore, DNA from a strain of *M. wenyonii* served as a positive control, while DEPC-treated water was utilized as a negative control in all PCR reactions. The PCR procedures were conducted as described in previous studies (43, 44). In addition, nested primers Rick-out/Rick-in for *R. felis* (45), Apla-sense and ECB primers for *Anaplasma* (46), HAM-1F and HPF-2R primers for *Hepatozoon* (47), and BTH 18S 1st F/R and BTH 18S 2nd F/R primers for *Babesia* and *Theileria* (48) were used to detect potential tick-borne pathogens associated with anemia in cats. The extracted blood and tick DNA were reconstituted in 50 μL of double-distilled water, and the quantity and quality of DNA were assessed using a spectrophotometer (UV1000, Techcomp, China). The PCR amplification reactions were performed using an EasyTaq® PCR SuperMix kit (TransGen, Beijing, China) in a total reaction of 20 μL, which included 10 μL of 2× EasyTaq® PCR SuperMix, 0.4 μM of each primer, and 20 ng template DNA. The amplification conditions were as follows: predenaturation at 94°C for 5 min, followed by 30 cycles of 30 s at 94°C, 30 s at the appropriate annealing temperature determined by the specific primers, 2 min at 72°C, and a final extension at 72°C for 10 min. The primers and annealing temperature are shown in Table 1.

**Table 1:** Primer sequences for pathogens.

Pathogens	Sequence (5′-3′)	Product size (bp)	Annealing temperature (°C)	Reference
Hemotropic mycoplasmas	H-MYC-F: ACGAAAGTCTGATGGAGCAAT(A/G)	170/190	60	Jensen et al. (43) and Shi et al. (30)
	H-MYC-R: ACGCCCAATAAATCCG(A/G)ATAAT			
*R. felis*	Rick-out-F: AGTAAATCCAATAATAAAAAATGCKCTTAATA	446	57	Zhang et al. (45)
	Rick-out-R: CTTAAAGATGAATATTTTATTGAGAGAAAAT			
	Rick-in-F: ATGAGCAGAATGCTTCTACTTCAACA	353	57	
	Rick-in-R: ATGAGCAGAATGCTTCTACTTCAACA			
*Anaplasma*	Apla-sense: -CTCAGAACGAACGCTGGCGGCAAGC	476	60	Santos et al. (46)
	ECB: CGTATTACCGCGGCTGCTGGC			
*Hepatozoon*	HAM-1F: GCCAGTAGTCATATGCTTGTC	1700	56	Hodžić et al. (10)
	HPF-2R: GACTTCTCCTTCGTCTAAG			
*Babesia/ Theileria*	BTH 18S 1 st-F: GTGAAACTGCGAATGGCTCATTAC	1,400 ~ 1,600	55	Masatani et al. (48)
	BTH 18S 1 st-R: AAGTGATAAGGTTCACAAAACTTCCC			
	BTH 18S 2 st-F: GGCTCATTACAACAGTTATAGTTTATTTG			
	BTH 18S 2 st-R: CGGTCCGAATAATTCACCGGAT			
*‘Ca.* M. haemobos*’*	MHBforw: GAA TTA ATG CTG ATG GTA TGC CTA A	1,393	52	Meli et al. (44)
	MHBrev: CCA ATC AGA ATG TTC ACT CTA GAT GC			

Following the initial molecular screening, all mycoplasma-positive amplicons were purified and sequenced, as previously described (14). The resulting sequences were then compared to relevant sequences available in the NCBI databases utilizing a BLAST search. Subsequently, longer fragments (1,393 bp) of the 16S rRNA gene were amplified from all samples that tested positive for *‘Ca.* M. haemobos*’* using the MHBforw and MHBrev primers (44). These fragments were also purified and sequenced following the aforementioned protocol.

### 2.5 Phylogenetic analysis

The sequences of the long 16S rRNA gene were compared and aligned using the CLUSTALW program, incorporating strains isolated from cattle, buffalo, sheep, ticks, and dogs from various regions worldwide. Subsequently, a phylogenetic analysis was performed using MEGA6 (49), employing the neighbor-joining criterion and the Kimura two-parameter model (11, 14, 22, 44, 50). The robustness of the hypothesis was tested using bootstrap analysis with 1,000 replicates.

### 2.6 Statistical analysis

In this study, cats were divided into two groups: Group 1 comprised 57 anemic cats that exhibited clinical signs and had hematological values below the normal reference ranges, while Group 2 consisted of 21 healthy cats with hematological values within the normal reference ranges. Statistical analysis for significant differences was performed between groups using SPSS 17 software (IBM, USA) on the chi-square test. *p*-value<0.05 was considered the threshold for statistical significance.

## 3 Results

### 3.1 Tick identification

In total, 640 ticks were collected from both cats and the environment. This collection included 284 adult ticks obtained from cats, along with 336 adult ticks and 20 nymph ticks collected from the environment. Following a thorough examination using morphological and taxonomic keys, a total of 119 male adult ticks and 501 female adult ticks were identified. Subsequent molecular identification through sequencing of the 12S rRNA gene revealed that these adult ticks comprised three species from two genera within the family Ixodidae: 407 *H. longicornis* (199 and 208 collected from cats and the environment, respectively), 155 *R. microplus* (58 and 97 collected from cats and the environment, respectively), and 78 *Rhipicephalus sanguineus sensu lato* (*R. sanguineus* sl) (27 collected from cats and 51 from the environment). Furthermore, both engorged and unengorged ticks were recorded among the three tick species collected from cats and the environment. Detailed information regarding the adult tick species, their source, sexes, life stages, and numbers is presented in Table 2.

**Table 2:** Species of ticks collected from cats and the environment in this study.

Species	Source		No. of ticks		
		Adult/Male (engorged)	Adult/Female (engorged)	Nymph	Total (engorged)
*Haemaphysalis longicornis*	Cats	38 (24)	161 (114)	0	199 (138)
	Environment	31 (1)	162 (6)	15	208 (7)
*Rhipicephalus microplus*	Cats	13 (10)	45 (36)	0	58 (46)
	Environment	22 (0)	72 (3)	3	97 (3)
*Rhipicephalus sanguineus sensu lato*	Cats	5 (3)	22 (14)	0	27 (17)
	Environment	10 (0)	39 (2)	2	51 (2)
Total		119 (38)	501 (175)	20	640 (213)

### 3.2 Pathogen identification in cats and ticks

As shown in Table 3, hemotropic mycoplasma infection rates were 54.4% (31 out of 57) and 14.3% (3 out of 21) in blood samples in Group 1 and Group 2, respectively; 55.4% (102 out of 184), 26.7% (4 out of 15), and 29.3% (61 out of 208) in *H. longicornis* in Group 1, Group 2, and the environment, respectively; 64.0% (32 out of 50), 25.0% (2 out of 8), and 35.0% (34 out of 97) in *R. microplus* in Group 1, Group 2, and the environment, respectively; and 68.2% (15 out of 22), 20.0% (1 out of 5), and 17.6% (9 out of 51) in *R. sanguineus* sl in Group 1, Group 2, and the environment, respectively. Significant differences in mycoplasma infection rates were observed between Group 1 and Group 2 in the blood samples (*p* = 0.002). All hemotropic mycoplasma-positive amplicons were sequenced and aligned using the BLAST search tool in GenBank. The results indicated the prevalence rates of *‘Ca.* M. haemominutum*’*, *M. haemofelis*, and *‘Ca.* M. haemobos*’* in the blood samples, as well as in samples from *H. longicornis*, *R. microplus*, and *R. sanguineus* sl. In addition, screening for other pathogens revealed the presence of *R. felis* in cats and three tick species, with no other pathogens detected. Moreover, two types of co-infections (*‘Ca.* M. haemominutum*’* + *R. felis* and *‘Ca.* M. haemobos*’* + *R. felis*) were identified in anemic cats, *H. longicornis*, and *R. sanguineus* sl.

**Table 3:** Frequency of tick-borne pathogens in cat and tick samples.

Samples	No.	Hemoplasmas (percentage)	*‘Ca. M. haemominutum’* (sum)^a^	*M. haemofelis* (sum)^b^	*‘Ca. M. haemobos’* (sum)^c^	*R. felis* (sum)^d^	*‘Ca. M. haemominutum’* + *R. felis*	*‘Ca. M. haemobos’* + *R. felis*
Blood (1. anemic)	57	31 (54.4%)	18	4	9	7	3	2
Blood (2. healthy)	21	3 (14.3%)	3	0	0	0	0	0
*H. longicornis* (1. anemic)	184	102 (55.4%)	68 (50)	12 (9)	22 (16)	19 (15)	6	3
*H. longicornis* (2. healthy)	15	4 (26.7%)	2 (2)	1 (1)	1 (1)	2 (2)	0	0
*H. longicornis* (En)	208	61 (29.3%)	30 (1)	13 (0)	18 (0)	22 (14)	5	1
*R. microplus* (1. anemic)	50	32 (64.0%)	11 (10)	8 (7)	12 (10)	4 (3)	0	0
*R. microplus* (2. healthy)	8	2 (25.0%)	0	0	2 (2)	0	0	0
*R. microplus* (En)	97	34 (35.0%)	13 (0)	1 (0)	20 (1)	2 (0)	0	0
*R. sanguineus* sl (1. anemic)	22	15 (68.2%)	7 (6)	2 (1)	6 (4)	5 (5)	0	1
*R. sanguineus* sl (2. healthy)	5	1 (20.0%)	0	0	1 (0)	1 (0)	0	0
*R. sanguineus* sl (En)	51	9 (17.6%)	4 (0)	0	5 (0)	2	0	0

^a^The number in the parentheses means the positive ‘*Ca*. M. haemominutum’ number detected in engorged ticks; ^b^The number in the parentheses means the positive *M. haemofelis* number detected in engorged ticks; ^c^The number in the parentheses means the positive ‘*Ca. M. haemobos*’ number detected in engorged ticks; ^d^The number in the parentheses means the positive *Rickettsia felis* number detected in engorged ticks; 1. anemic: Group 1; 2. healthy: Group 2; En: Environment. ‘*Ca*. M. haemominutum’: ‘*Candidatus* Mycoplasma haemominutum; ’*M. haemofelis, Mycoplasma haemofelis; ‘Ca. M. haemobos’, Candidatus Mycoplasma haemobos; R. felis, Rickettsia felis*.

Initially, the infection rates of *‘Ca.* M. haemominutum*’* were analyzed. *‘Ca.* M. haemominutum*’* infection rates were 31.6% (18 out of 57) and 14.3% (3 out of 21) in the blood samples in Group 1 and Group 2, respectively, and no significant differences (*p* = 0.127) were detected. In contrast, when comparing the infection rates in *H. longicornis* from the environment group (14.4%, 30 out of 208), a significant difference (*p* < 0.001) was noted in Group 1 (37.0%, 68 out of 184), whereas no significant difference (*p* = 1.000) was observed in Group 2 (13.3%, 2 out of 15). Furthermore, when compared to the infection rate of *R. microplus* in the environment group (13.4%, 13 out of 97), the infection rates of *R. microplus* in both Group 1 (22.0%, 11 out of 50) and Group 2 (0%, 0 out of 8) did not exhibit significant differences (*p* = 0.095 and *p* = 0.595, respectively). In addition, when comparing the infection rates of *R. sanguineus* sl in the environment group (7.8%, 4 out of 51), a significant difference (*p* = 0.023) was found in Group 1 (31.8%, 7 out of 22), whereas no significant difference (p = 1.000) was observed in Group 2 (0%, 0 out of 5).

In the second analysis, the infection rates of *‘Ca.* M. haemobos*’* were compared across different groups. *‘Ca.* M. haemobos*’* infection rates were 15.8% (9 out of 57) and 0% (0 out of 21) in the blood samples in Group 1 and Group 2, respectively, and no statistically significant differences (*p* = 0.124) were observed. Furthermore, when compared to the infection rate of *H. longicornis* in the environment group (8.7%, 18 out of 208), no significant differences (*p* = 0.281 and p = 1.000, respectively) were observed in Group 1 (12.0%, 22 out of 184) and Group 2 (6.7%, 1 out of 15). Similarly, the infection rates of *R. microplus* in Group 1 (24.0%, 12 out of 50) and Group 2 (25.0%, 2 out of 8) showed no significant differences (*p* = 0.638 and p = 1.000, respectively) compared to the environment group (20.6%, 20 out of 97). In addition, when comparing the infection rate of *R. sanguineus* sl in the environment group (9.8%, 5 out of 51), no significant differences (*p* = 0.119 and *p* = 0.445, respectively) were found in Group 1 (22.7%, 5 out of 22) and Group 2 (20.0%, 1 out of 5). Notably, *‘Ca.* M. haemobos*’-*positive samples, which included one *H. longicornis*, two *R. microplus*, and one *R. sanguineus* sl, were collected from negative cats in Group 2.

*M. haemofelis* infection rates were 7.0% (4 out of 57) and 0% (0 out of 21) in the blood samples in Group 1 and Group 2, respectively; 6.5% (12 out of 184), 6.7% (1 out of 15), and 6.3% (13 out of 208) in *H. longicornis* in Group 1, Group 2, and the environment, respectively; 16.0% (8 out of 50), 0% (0 out of 8), and 1.0% (1 out of 97) in *R. microplus* in Group 1, Group 2, and environment, respectively; and 9.1% (2 out of 22), 0% (0 out of 5), and 0% (0 out of 51) in *R. sanguineus* sl in Group 1, Group 2, and the environment, respectively. Additional details are provided in Table 3. It is important to highlight that *‘Ca.* M. haemominutum*’*, *M. haemofelis*, *‘Ca.* M. haemobos*’*, and *R. felis* were detected in both engorged and un-engorged ticks across all three species, as presented in Table 3.

### 3.3 Sequence analysis of *‘ca.* M. Haemobos*’*

**Figure 1:**
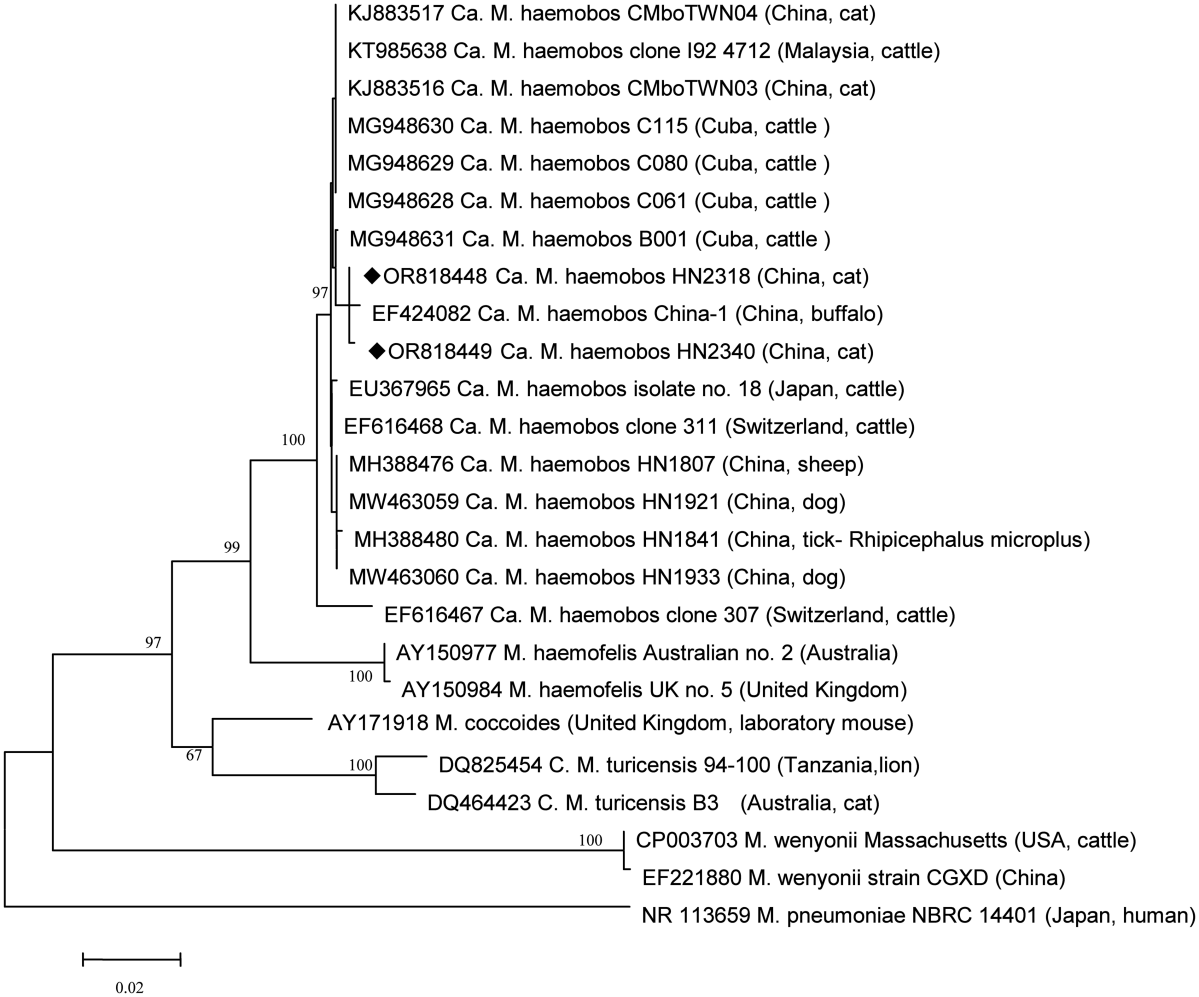
Phylogenetic analysis of *‘Ca.* M. haemobos*’* from cats in Henan Province, central China, and reference strains using the 16S rRNA sequences. New isolates in this study are highlighted with a symbol (◆).

For further analysis, the longer amplicons from all positive samples of *‘Ca.* M. haemobos*’* were amplified, recovered, and sequenced. A total of six different sequences were identified among these positive samples. Six strains were selected as representatives for detailed analysis: the HN1807 strain (GenBank Accession Number MH388476) and the HN1841 strain (GenBank Accession Number MH388480), which have been previously described in sheep and *R. microplus* (14); the HN1921 strain (GenBank Accession Number MW463059) and the HN1933 strain (GenBank Accession Number MW463060), which have been previously described in dogs (16); and two novel sequence types: HN2318 (GenBank Accession Number OR818448) and HN2340 (GenBank Accession Number OR818449). In addition, new sequences were observed in those three tick species. Specifically, the following *‘Ca.* M. haemobos*’* strains were included in the analysis: clones 307 (EF616467) and 311 (EF616468) (cattle, Switzerland); no. 18 (EU367965) (cattle, Japan); C115, C080, C061, and B001 (MG948630) (cattle, Cuba); I924712 (KT985638) (cattle, Malaysia); HN1804 (MH388478) (tick, China); HN1807 (MH388476) (sheep, China); HN1823 (MH388475) (goat, China); HN1921 (MW463059) (dog, China); and *CMbo*TWN03 (KJ883516) and *CMbo*TWN04 (KJ883517) (cat, China). Furthermore, for the purpose of phylogenetic analysis, the following isolates were included: *M. haemofelis* isolates Australian no. 2 (AY150977) (Australia) and UK no. 5 (AY150984) (United Kingdom); *M. coccoides* (AY171918) (United Kingdom, laboratory mouse); *Ca.* M. turicensis 94–100 (DQ825454) (Tanzania, lion) and B3 (DQ464423) (Australia, cat); *M. wenyonii* Massachusetts (CP003703) (USA, cattle) and CGXD (EF221880) (China); and *M. pneumoniae* NBRC 14401 (NR 113659) (Japan, human). The two new representative isolates were compared to other *‘Ca.* M. haemobos*’* strains available in GenBank, revealing that the 16S rRNA sequences of the two new isolates exhibited 99.34–99.57% identity with those of other isolates. A phylogenetic tree was inferred based on the 16S rRNA sequence (Figure 1), indicating that the two new representative isolates clustered within the species of *‘Ca.* M. haemobos*’*. Moreover, the two new isolates identified in the present study were found to be most closely related to an isolate (China-1) (EF424082) from buffalo in China, while being most distantly related to the isolate from Switzerland (clone 307).

## 4 Discussion

To date, no studies focusing on *‘Ca.* M. haemobos*’* in cats have been reported in central China. Given the limited evidence regarding the association between *‘Ca.* M. haemobos*’* and infections in cats, this study aimed to clarify this issue. Although no significant difference in infection rates was observed between anemic cats and healthy cats, the potential association between *‘Ca.* M. haemobos*’* and anemic syndrome in cats cannot be excluded. The presence of other pathogens in the anemic cats and the limited sample size in this study may constrain our analysis and conclusions. Further research involving experimental infections in cats is warranted to elucidate the relationship between ‘Candidatus Mycoplasma haemobos’ and feline health. In Henan Province in central China, *‘Ca.* M. haemobos*’* has been identified in blood samples collected from sick cattle and goats with a rate of 63.9% (23 out of 36) and 58.2% (32 out of 55) (15), blood samples from sick sheep with a rate of 40.0% (10 out of 25) (51), and blood samples from healthy dogs and anemic dogs with the rates of 28.6% (4 out of 14) and 63.4% (26 out of 41), respectively. This study represents the first report of *‘Ca.* M. haemobos*’* in cats within the country, and the prevalence rate in cats is not much different than in other species. Phylogenetic analysis revealed that the two new isolates identified in this study are not most closely related to the isolates found in goats, sheep, dogs, and ticks in the region described in previous research (14–16), but rather to an isolate from buffalo in China (52). Two possible explanations for this finding included the frequent importation and trading of livestock in China, which may facilitate the spread of *‘Ca.* M. haemobos*’* to new areas, or the potential evolution of the pathogen as it adapts to new hosts or vectors, such as cats and ticks. Previous studies have indicated that *R. microplus* and *H. longicornis* can harbor *‘Ca.* M. haemobos*’* and are associated with its transmission in goats, cattle, and dogs. Given that the cats in this study share their habitat with these animals and that these ticks can infest cats, the role of ticks in the transmission of *‘Ca.* M. haemobos*’* to cats remains unclear. Therefore, an investigation was conducted on all ticks parasitizing cats and those present in the environment to address this question.

In Hungary, 21 cattle were diagnosed as positive for *‘Ca.* M. haemobos*’*, and four species of ticks were collected from these animals: *Dermacentor reticulatus*, *Haemaphysalis inermis*, *Ixodes ricinus*, and *Dermacentor marginatus.* However, all ticks were negative for *‘Ca.* M. haemobos*’* (53). This study identified three species of ticks. In addition to *R. microplus* and *H. longicornis* ticks, which have been documented on goats, cattle, and dogs (14–16), *R. sanguineus* sl ticks were newly identified as carriers of *‘Ca.* M. haemobos*’.* Notably, *‘Ca.* M. haemobos*’*-positive ticks were detected on both positive and negative cats, including ticks that were not engorged. This finding is consistent with previous research, indicating that the eggs of *R. microplus* can acquire *‘Ca.* M. haemobos*’* from female ticks and retain the pathogen during development stages (30). Consequently, it is plausible that the tick collected from a negative cat was indeed positive for the pathogen. Although the presence of *‘Ca.* M. haemobos*’* in *H. longicornis* ticks has been reported in earlier studies (15, 16), the potential for transovarial transmission of *‘Ca.* M. haemobos*’* by female *H. longicornis* ticks remains to be elucidated. Given the high positive rates of *‘Ca.* M. haemobos*’* in *H. longicornis* ticks associated with dogs (24 out of 150) (16), cattle (20 out of 45), goats (6 out of 16) (15), and cats in this study (41 out of 407), it is crucial to investigate whether *H. longicornis* ticks can act as vectors during their developmental stages and to conduct experimental transmission studies of *‘Ca.* M. haemobos*’* to potential hosts such as goats, cattle, dogs, and cats in future research. To date, *R. sanguineus* sl ticks have been identified on cattle, goats, and dogs (54), as well as on cats in four provinces (Hebei, Anhui, Zhejiang, and Guangxi) in China. However, their presence on cats in Henan Province had not been previously reported (55). This study confirms the occurrence of *R. sanguineus* sl on cats in this region for the first time. While numerous pathogens have been detected in *R. sanguineus* sl (34), the presence of *‘Ca.* M. haemobos*’* in this tick had not been documented until this study. Among the positive ticks, one was collected from a negative cat. Considering that *R. sanguineus* sl is a three-host tick (56), this positive tick may have acquired *‘Ca.* M. haemobos*’* from a previous host during blood feeding or may have carried the pathogen from an earlier development stage, such as from eggs. In either scenario, *‘Ca.* M. haemobos*’* could persist in *R. sanguineus* sl ticks for a certain duration. Furthermore, other livestock species, such as rabbits, pigs, and horses, also serve as hosts for the aforementioned ticks (57, 58). The potential for these animals to become infected with *‘Ca.* M. haemobos*’* while infested with ticks remains unknown, and future investigations should be carried out to determine the prevalence of *‘Ca.* M. haemobos*’* across various livestock species. Furthermore, no statistically significant differences (*p* > 0.05) were observed in the infection rates of *‘Ca.* M. haemobos*’* among three tick species in cats from both Group 1 and Group 2 when compared to the infection rates of *‘Ca.* M. haemobos*’* in the environment group. In addition, the majority of positive ticks collected from the environment were not engorged. These findings suggested that the presence of ‘*Ca.* M. haemobos*’* in ticks collected from cats may not be attributable to the parasite acquired through a blood meal. It is possible that *‘Ca.* M. haemobos*’* can persist in the three tick species for a certain duration before or after infesting the host animals.

In addition to *‘Ca.* M. haemobos*’*, *‘Ca.* M. haemominutum*’*, *M. haemofelis*, and *R. felis* were also detected in cats, *H. longicornis*, *R. microplus*, and *R. sanguineus* sl. Specifically, infections with *‘Ca.* M. haemominutum*’* and *M. haemofelis* in cats have been reported with prevalence rates of 3.4 and 0.9% in 668 client-owned cats in Beijing and Shanghai, China, respectively (36). Of those 668 cats, 131 were anemic with a hemotropic mycoplasma infection rate of 9.2%. Furthermore, the prevalence rates in Shanghai and Beijing were all lower than those in Henan in this study. Several factors may explain this difference: 668 cats in Shanghai and Beijing were housed in the city and had less exposure to the wild; blood samples were collected not only in the summer season and nearly half of the cats were using ectoparasiticides. In addition, the study did not record whether the positive cats had been infested by ticks. In Iran, 361 blood samples were collected from healthy cats for hemotropic mycoplasma screening; the results showed that the rates of *‘Ca.* M. haemominutum*’* and *M. haemofelis* were 10.5 and 2.2%, respectively (59). Similarly in Brazil, *‘Ca.* M. haemominutum*’* and *M. haemofelis* were 8.9 and 4.4%, respectively, in 45 healthy stray cats (60). These positive rates are similar to those in healthy cats in our study. In Romania, *‘Ca.* M. haemominutum*’* and *M. haemofelis* were 15.7 and 5.9%, respectively, in 51 unhealthy cats (61). These positive rates are lower than those in anemic cats in our study. Based on the above studies, it is indicated that the infection rate of *Mycoplasma haemofelis* in cats may be related to factors such as countries, regions, the health status of cats, feeding methods, and the season of sample collection.

In northern Switzerland, feline hemotropic mycoplasmas were identified in several *Ixodes* sp. (2.8%, 2 out of 71) and *Rhipicephalus* sp. ticks (4.3%, 1 out of 23) collected from animals (62). In Italy, *Ixodes ricinus* and *Ixodes trianguliceps* ticks (0.6%, 3 out of 50) were found to be positive for *‘Ca.* M. haemominutum*’*; meanwhile, *Ixodes trianguliceps* (0.2%, 1 out of 540) was also found to be positive for *M. haemofelis* (63)*. In Japan, among eight pools of* unfed *Ixodes ovatus* ticks collected from vegetation, three pools were positive for *‘Ca.* M. haemominutum*’* (64), and, similarly, *Ixodes tanuki* ticks (3.3%, 1 out of 30) collected from Tsushima leopard cats were also found to carry this pathogen (65). These studies suggest that ticks have the potential to serve as carriers for feline hemotropic mycoplasmas. These positive rates are lower than those in the three tick species in our study. However, in Italy, one study showed that no hemotropic mycoplasmas have been detected in 17 *R. sanguineus* sl tick samples collected from cats (66). To the best of our knowledge, neither *‘Ca.* M. haemominutum*’* nor *M. haemofelis* has been documented in *H. longicornis* or *R. microplus* ticks in China. Our research demonstrates significant differences in the infection rates of *‘Ca.* M. haemominutum*’* between Groups 1 and 2 for *H. longicornis* and *R. sanguineus* sl. These findings suggest that these tick species may serve as potential vectors for this mycoplasma in cats, and that *‘Ca.* M. haemominutum*’* should be associated with anemia in cats in Group 1. Further studies should evaluate the competence of these ticks in transmitting ‘*Ca. M.* haematobium’ to cats. *R. felis* has been detected in *Ixodes granulatus* ticks from rodents (67) and *R. sanguineus* sl ticks from dogs (68) in Taiwan. In addition, *Rickettsia* spp. have been identified in *H. longicornis* and *R. microplus* ticks from non-cat hosts, as well as through flagging over vegetation in Jiangxi province, located in southeastern China (69). In Jiangsu province, *R. felis* was first identified in *R. sanguineus* sl ticks (45). To date, studies on *R. felis* infections in cats in China remain limited. Our findings suggest that the prevalence of *R. felis* should not be overlooked in backyard farms in China, particularly as the presence of these ticks on infected cats may increase the public health risk for individuals in agricultural settings.

## 5 Conclusion

This study represents the first investigation of hemoplasmas *Mycoplasma* in cats and ticks in central China. In addition to *‘Ca.* M. haemominutum*’* and *M. haemofelis, ‘Ca.* M. haemobos*’* was first detected in cats. ‘*Ca.* M. haemominutum’ appears to be associated with anemic syndrome in cats, while further research is warranted to explore the relationship between ‘*Ca.* M. haemobos’ and clinical signs in felines. Specifically, two types of ‘*Ca.* M. haemobos’ sequences have been detected both in ticks and cats. Meanwhile, these three hemotropic mycoplasmas were also found in the parasitic ticks and questing ticks, including *H. longicornis*, *R. microplus*, and *R. sanguineus* sl. Among these findings, *‘Ca.* M. haemominutum*’* and *M. haemofelis* in *H. longicornis* and *R. microplus*, and ‘*Ca.* M. haemobos’ in *R. sanguineus* sl. were first documented in China. In the future, transmission experiments are needed to investigate the capacity for transmitting hemoplasmas *Mycoplasma* among animals by these ticks.

## Data Availability

The datasets presented in this study can be found in online repositories. The names of the repository/repositories and accession number(s) can be found in the article/Supplementary material.

## Supplementary material

Supplementary Figure 1 The results of PCR analysis of the partial 16S rRNA gene of hemotropic mycoplasmas conducted on some blood and tick samples. The DNA marker is represented by the letter M, while negative and positive controls are indicated by “-” and “+”. Blood and tick samples numbered 1 through 22 were analyzed.

Supplementary Figure 2 The results of PCR analysis of the long 16S rRNA gene of ‘*Ca*. M. haemobos’ conducted on some blood and tick samples that tested positive for ‘*Ca*. M. haemobos’ by sequencing. The DNA marker is represented by the letter M, while negative control is indicated by “-”. Positive blood and tick samples numbered 1 through 23 were analyzed.

Supplementary Table 1 Isolates, organism, host and GenBank number of hemotropic mycoplasmas samples.
