# Supplementary figures and images for: Molecular investigation reveals three hemotropic mycoplasmas in cats and three tick species in China
Source: Front Vet Sci. 2025 Jan 30;12:1522904. doi: 10.3389/fvets.2025.1522904 (PMC11821626; doi:10.3389/fvets.2025.1522904)

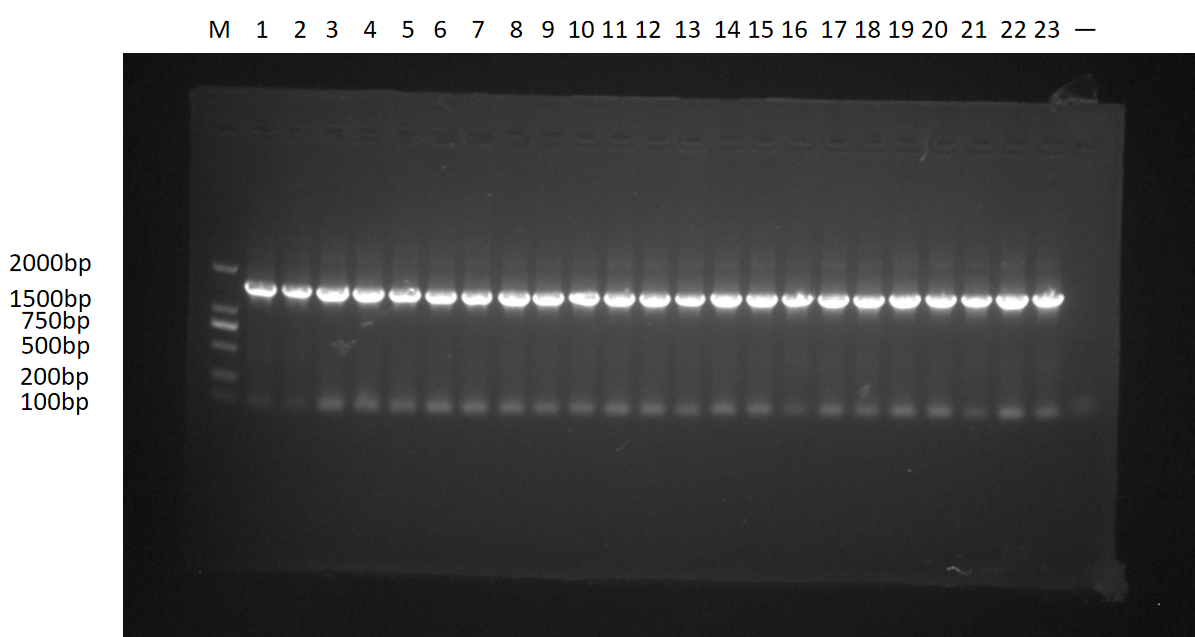

Supplement: Supplementary Figure 1 — The results of PCR analysis of the partial 16S rRNA gene of hemotropic mycoplasmas conducted on some blood and tick samples. The DNA marker is represented by the letter M, while negative and positive controls are indicated by “-” and “+”. Blood and tick samples numbered 1 through 22 were analyzed. [file Data_Sheet_1.ZIP › Figure 2.tif]

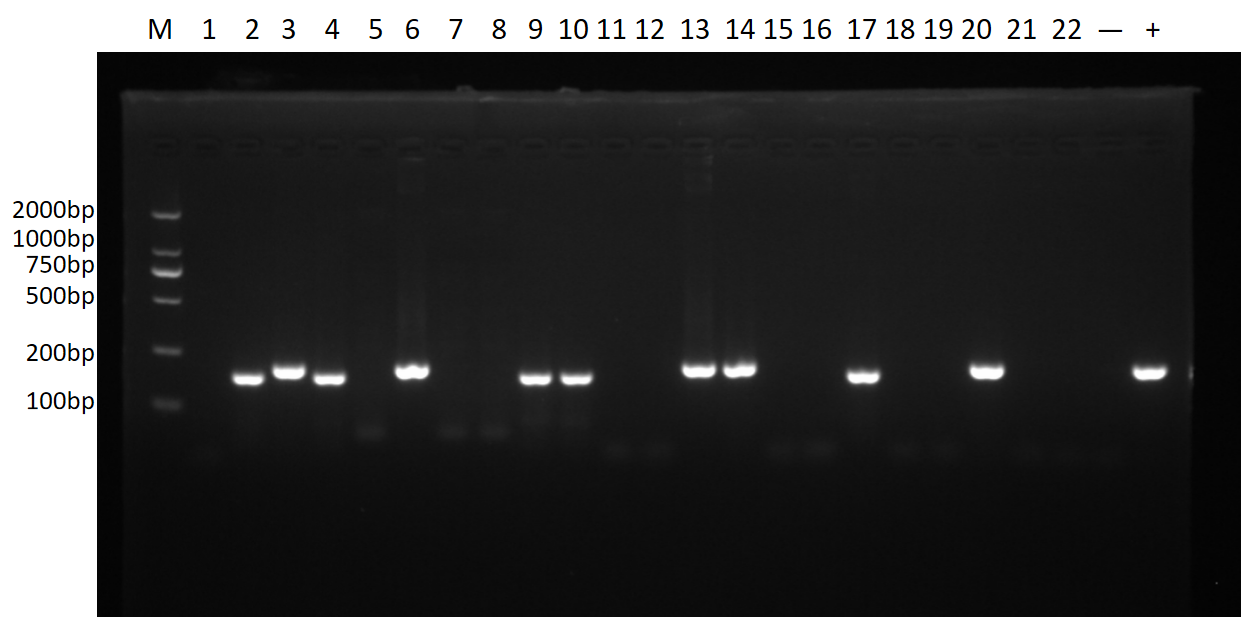

Supplement: Supplementary Figure 1 — The results of PCR analysis of the partial 16S rRNA gene of hemotropic mycoplasmas conducted on some blood and tick samples. The DNA marker is represented by the letter M, while negative and positive controls are indicated by “-” and “+”. Blood and tick samples numbered 1 through 22 were analyzed. [file Data_Sheet_1.ZIP › Figure 1.tif]
